# Unravelling the genetic basis of simplex Retinitis Pigmentosa cases

**DOI:** 10.1038/srep41937

**Published:** 2017-02-03

**Authors:** Nereida Bravo-Gil, María González-del Pozo, Marta Martín-Sánchez, Cristina Méndez-Vidal, Enrique Rodríguez-de la Rúa, Salud Borrego, Guillermo Antiñolo

**Affiliations:** 1 Department of Genetics, Reproduction and Fetal Medicine, Institute of Biomedicine of Seville, University Hospital Virgen del Rocío/CSIC/University of Seville, Seville, Spain; 2 Centre for Biomedical Network Research on Rare Diseases (CIBERER), Seville, Spain; 3 Department of Ophthalmology, University Hospitals Virgen Macarena and Virgen del Rocío, Seville, Spain; 4 Retics Patologia Ocular, OFTARED, Instituto Salud Carlos III, Madrid, Spain.

## Abstract

Retinitis Pigmentosa (RP) is the most common form of inherited retinal dystrophy (IRD) characterized ultimately by photoreceptors degeneration. Exhibiting great clinical and genetic heterogeneity, RP can be inherited as an autosomal dominant (ad), autosomal recessive (ar) and X-linked (xl) disorder. Although the relative prevalence of each form varies somewhat between populations, a major proportion (41% in Spain) of patients represent simplex cases (sRP) in which the mode of inheritance is unknown. Molecular genetic diagnostic is crucial, but also challenging, for sRP patients because any of the 81 RP genes identified to date may be causative. Herein, we report the use of a customized targeted gene panel consisting of 68 IRD genes for the molecular characterization of 106 sRP cases. The diagnostic rate was 62.26% (66 of 106) with a proportion of clinical refinements of 30.3%, demonstrating the high efficiency of this genomic approach even for clinically ambiguous cases. The high number of patients diagnosed here has allowed us to study in detail the genetic basis of the sRP. The solved sRP cohort is composed of 62.1% of arRP cases, 24.2% of adRP and 13.6% of xlRP, which implies consequences for counselling of patients and families.

Retinitis Pigmentosa (RP [MIM 268000]) is the most prevalent form of inherited retinal dystrophy (IRD) affecting 1:3,000 to 4,000 individuals worldwide^1^. Typical RP is characterized by early loss of rod photoreceptors, manifesting with initial night blindness and tunnel vision, followed by the secondary loss of cone photoreceptors leading to decreased visual acuity and macular affectation^2^. However, the age of onset, disease progression and severity of symptoms can vary markedly among patients, even within the same family^1^. RP is a genetic disorder inherited as Mendelian traits in most cases. The frequencies of autosomal dominant (adRP), autosomal recessive (arRP) and X-linked RP (xlRP) are reported to be 12%, 39% and 4%, respectively, in Spain^3^. Although the relative prevalence of each form varies somewhat between populations, a major proportion (40% or higher) of patients represent isolated or simplex cases (sRP)^4^. Other types of non-Mendelian inheritance as mitochondrial inheritance^5^, digenism^6^ and uniparental isodisomy^7^ have also been described in RP. RP is also a highly heterogeneous genetic disorder, with mutations in more than 80 genes and loci identified to date (http://www.sph.uth.tm.edu/Retnet/ accessed May 2016).

Simplex or isolated cases are defined as those non consanguineous affected individuals with no affected first-degree relatives (parents, siblings, and children) and no reports of more distant affected family members. Although clinical examination of extended family members can often be revealing, the genetic approach of sporadic cases is complex. Traditionally, sRP cases have been considered recessive cases, with unaffected carrier parents. This is certainly true in many cases, but there are other possibilities. Perhaps, one parent is a carrier but the other mutation is *de novo*. Alternatively, it is estimated that *de novo* mutations in dominant inheritance genes are responsible for at least 1–2% of isolated cases^4^ and at least 15% of affected males of sRP harboured mutations in the xlRP genes *RPGR* and *RP2*^8^. Moreover, incomplete penetrance, or nonpenetrance, in prior generations can also confuse the picture of sRP^9^. All these concerns can be confusing for the patient, who may require a more detailed explanation and genetic counselling, and in some cases, it may be even necessary to redefine the family disease.

In this regard, molecular genetic testing is crucial, but also challenging, for sRP patients, who can carry causative mutations in any of the 81 known RP genes. The next-generation sequencing (NGS) technologies introduced in the last few years have shown to be very helpful in the molecular diagnosis of IRD families^10^. Moreover, the development of population specific targeted gene panels allows a more efficient interpretation of sequence variations and overcomes limitations associated with the large amount of data generated by higher performance platforms^11^,^12^.

Herein, we report an in-house targeted gene panel approach to uncover the genetic cause of 106 sRP Spanish families. The diagnostic rate was 62.26% (66 of 106), demonstrating the high efficiency of this genomic approach. Our data revealed that the solved sRP cohort is composed of 62.1% of arRP cases, 24.2% of adRP and 13.6% of xlRP, which implies consequences for counselling of patients and families. These findings represent a significant advance in efforts to unravel the genetic basis of sRP.

## Results

### Clinical features

All index patients included in this study received an initial clinical diagnosis of simplex RP. However, due to the high clinical heterogeneity of this disease, diverse clinical manifestations were found (Supplementary Table S1 and data not shown). The majority of cases (~70%) showed typical RP with initial night blindness and at least two of the classic fundus findings (bone-spicule pigmentation, narrowed vessels and pale optic disc). The remaining cases presented different degrees of macular involvement and other initial symptoms (reduced visual field or decreased visual acuity). The age of onset and the progression of the disease were variable among families and additional findings such as photophobia, myopia, cataracts, dischromatopsia and nystagmus were found in ~45% of cases.

### Reliability of NGS data

The reliability of NGS data depend on three criteria: depth of coverage, heterogeneity and accuracy of sequencing. On average, sequencing and mapping resulted in a mean coverage of 742.1x and a percentage of reads mapping on target of 96.7%. Within the targeted region, ~95% of the bases were covered >100x and ~65% >500x, which indicated that sufficient coverage was achieved for sensitive detection of variants. Application of variant calling allowed obtaining a mean of 847 raw variants per sample, which were filtered and annotated to identify causative mutations. Variants present in the two positive-control samples were successfully detected after the bioinformatics analysis, demonstrating the diagnostic accuracy of this approach.

### Identification and assessment of candidate variants

Application of the data analysis pipeline allowed the identification of 96 potential causative mutations in 66 out of the 106 families (62.26%) (Table 1). However, given the difficulty of obtaining samples from additional relatives in sporadic cases in which there is only one affected member, we could perform segregation analysis in only 21 out of 66 families. Most mutations in the remaining cases were considered as the likely disease cause since they were consistent with previously reported genotype-phenotype correlations and met the established pathogenicity criteria (see Material and Methods section). Seventeen of these families need additional studies to achieve a full diagnosis since the nature of the mutation (synonymous and possible dominant *de novo* variants) and the absence of available family members did not allow us to complete the required studies to obtain a reliable estimation of their pathogenicity. Still, a minimum diagnostic rate of 46.22% was achieved by the application of this IRD panel. Remarkably, the majority of the *in silico* splicing prediction tools showed that all these synonymous and splicing variants were probably damaging. Of note, patients will not be informed until segregation studies confirm the pathogenicity of the detected variants.

**Figure 1:**
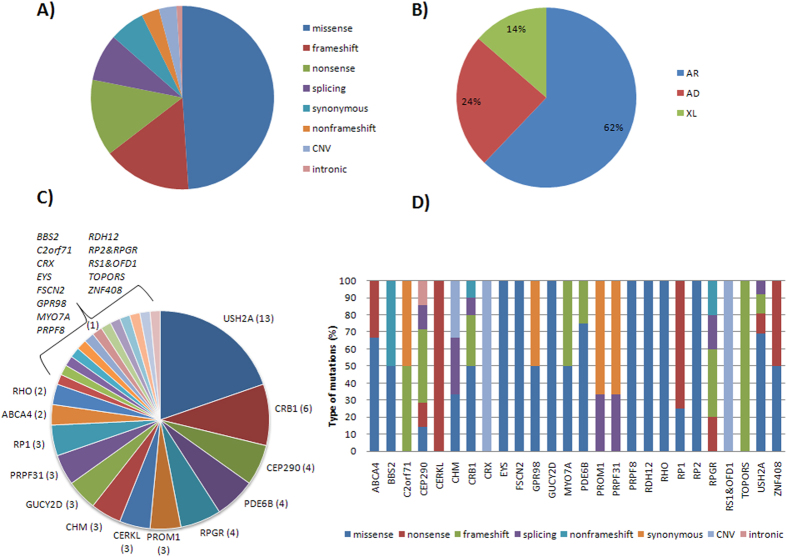
Prevalence analyses of solved cases. (**A**) Proportion of each type of mutation. (**B**) Principal detected modes of inheritance. (**C**) Recurrence of all mutated retinal genes. (**D**) Distribution of different types of mutations for each of the identified mutated gene.

Detected candidate variants included 3 CNVs, 47 missense, 6 synonymous, 13 nonsense, 15 frameshift, 3 nonframeshift, 1 intronic and 8 splicing mutations (Fig. 1A). Thirty three of these variants were novel and absent in public databases (Table 1). Specifically, we found homozygous or compound heterozygous mutations in autosomal recessive IRD disease genes in 41 cases, heterozygous mutations in autosomal dominant IRD-associated genes in 16 cases and mutations in X-linked disease genes in 9 cases (Fig. 1B). The most frequent mutated gene for autosomal recessive cases of this study was *USH2A*, for autosomal dominant cases was *PRPF31* and for X-linked cases was *RPGR* (Fig. 1C). In addition, although most causative mutations were specific to one family, we found recurrence for 6 of them, including: p.C759F of *USH2A* (5 cases), p.R303H of *USH2A* (3 cases), p.R257* of *CERKL* (3 cases), p.E767fs of *USH2A* (2 cases), p.I205fs of *CRB1* (2 cases) and p.S542* of *RP1* (2 cases). Finally, the proportion of type of mutations for each of the mutated genes is shown in Fig. 1D.

### Clinical refinements and genotype-phenotype correlations

**Figure 2:**
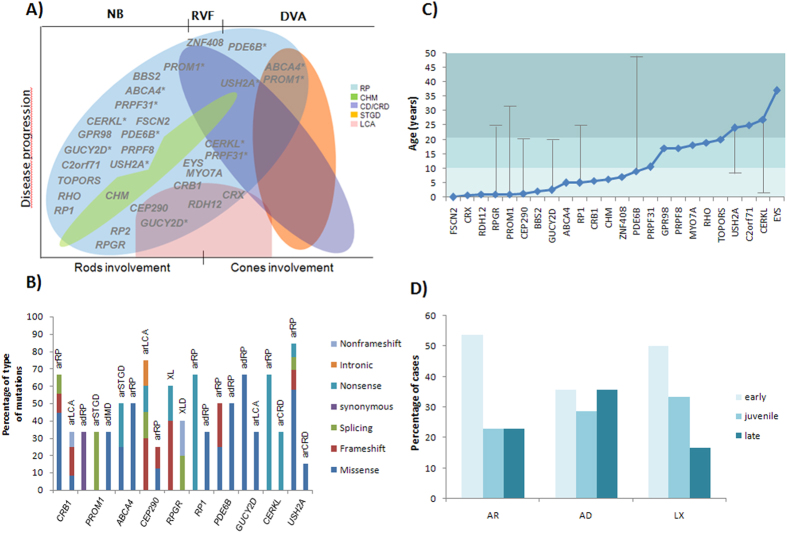
Genotype-phenotype correlations. (**A**) Overlapping phenotypes among different forms of non syndromic IRD: RP (Retinitis Pigmentosa), CHM (Choroideremia), CD/CRD (Cone dystrophy/Cone-rod dystrophy), STGD (Stargardt disease) and LCA (Leber congenital amaurosis). Gene positions show the associated phenotype/s and the first reported symptom of cases in which they are involved (NB: Night blindness; RVF: Reduced visual field; DVA: Decreased visual acuity). Asterisks indicate genes represented more than once in the figure due to the high heterogeneity. (**B**) Distribution of types of mutations behind the different phenotypes caused by the same gene. (**C**) Mean age of onset of each of the identified genes. Vertical lines show the age of onset of cases not included in the average to be considerably different from the rest. Of note, the gene *RHO* was identified in only two cases with very different clinical features hence the calculated average may not be representative. Moreover, this representation do not include clinical and genetic data of family #65 since the large deletion affects several genes and it is not possible to determine the involvement of each of them. Light blue marks early-onset cases while a juvenile onset is stated with medium blue and a late-onset with dark blue. (**D**) Distribution of early-, juvenile- and late-onset IRD for the different modes of inheritance.

After clinical reassessment, almost one-third of the cases showed clinical characteristics inconsistent with RP, which led to clinical diagnosis refinements taking into account the obtained genotypes. Thus, the diagnosis was modified from RP to Leber Congenital Amaurosis (LCA) in 7 cases, to choroideremia (CHM) in 3 cases, to cone-rod dystrophy (CRD) in 5 cases, to Stargardt disease (STGD) in 2 cases, to Usher syndrome (USH) in 2 cases and to macular dystrophy in 1 case (Supplementary Table S1). These results revealed that the considerable variation in clinical expression and phenotypic overlapping of single IRD entities hamper an accurate clinical diagnosis (Fig. 2A).

Although IRD-associated genes have been extensively studied so far, genotype-phenotype correlations are incompletely understood since many different genes may result in a similar disease phenotype and multiple clinical features may be caused by mutations in the same gene. In this regard, we found that mutations in *CRB1, CEP290, PROM1, CERKL, USH2A, GUCY2D* and *ABCA4* genes were involved in several disease phenotypes, and genes *RPGR, PDE6B* and *RP1* were acting under different modes of inheritance (Fig. 2A and B). Furthermore, our data have shown a relationship between the age of onset of symptoms and the disease-causing gene (Fig. 2C). Likewise, the proportion of early, juvenile and late IRD for each inheritance mode is shown in Fig. 2D.

Finally, this study has revealed interfamilial phenotypic variability involving the known pathogenic mutations p.R1129L of *ABCA4* and p.R257* of *CERKL*. The mutation p.R1129L of *ABCA4* was previously reported as causative of Stargardt disease but we found this mutation in homozygosis in the affected member of family #84, diagnosed of RP. Considering the presence of phenotype modifiers, we reanalyzed the NGS data and the recessive pathogenic *RP1* mutation p.R396* was found in heterozygosis. Secondly, we identified the homozygous disease-causing *CERKL* mutation p.R257* in three unrelated cases (#2, #53 and #55). This mutation is commonly associated with typical RP but the affected member of family #53 showed substantial macular involvement absent in the other two cases. No further variants were found by data reanalysis.

### Unsolved cases and variants of uncertain significance

We did not detect sequence alterations that completely explain the disease phenotype in 40 index patients (37.7%). In six cases, we found missense variants predicted as pathogenic but their high frequency in control populations led us to discard them as causative mutations. In addition, we identified potential pathogenic variants in 19 cases, including two heterozygous CNVs in *EYS*, five heterozygous pathogenic mutations in *ABCA4*, two heterozygous missense variants in *GPR98* and *CEP290,* and eight heterozygous mutations in *BBS12, CNGB3, NR2E3, PCDH15, PROM1, RP1, RPGRIP1* and *USH2A*, respectively (Supplementary Table S2). However, these mutations did not explain the disease by themselves and no second allele was found. In two other cases we found the combination of heterozygous exonic variants and heterozygous novel intronic variants that could be causative in compound heterozygosity. Nevertheless, these intronic mutations were categorized as unclear since further studies would be needed to evaluate their pathogenic role. Moreover, we detected two potential pathogenic mutations in *EYS* that did not segregate with the disease in one family. Finally, no candidate variants were found in the remaining 12 cases.

## Discussion

In this study, we performed targeted sequencing of 68 retinal genes to assist in the molecular diagnosis of 106 sRP Spanish cases, which resulted in a pathogenic mutation-detection rate of 62.26%. This yield is comparable to other studies involving capture panels covering a higher number of IRD genes^13^, which further demonstrate that increasing the number of genes does not necessarily improve the diagnostic rate for a particular population, hence a population-specific gene panel is the most cost-efficient option^11^. Unsolved cases will be subjected to whole exome sequencing (WES) and/or whole genome sequencing (WGS) to investigate their underlying genetic defect that may be in one of the retinal genes not included in the panel design, in a deep intronic region (supported by the high proportion of cases with only one pathogenic allele that does not explain the disease itself), in a new gene that has never been implicated in IRD or even follow other types of non-Mendelian inheritance. Therefore, targeted sequencing of selected IRD genes should be the primary choice in a NGS-based diagnostic routine for IRD and represents a powerful resource for selecting cases likely to carry mutations in new candidate genes^14^.

Almost 50% of RP patients are simplex cases with no family history of disease or consanguinity which makes it difficult to determine the mode of inheritance^15^. These cases may be caused by autosomal recessive, autosomal dominant (*de novo* and/or with incomplete penetrance) or X-linked gene mutations. Here, we found mutations following an autosomal recessive mode of inheritance in 62% of solved cases, which is in line with the traditional idea that most simplex cases are recessive with unaffected carrier relatives. However, mutations in the remaining 38% are consistent with an autosomal dominant (24%) or X-linked (14%) inheritance, which is higher than reported in previous studies^8^,^16^. Further analysis would be needed to confirm the pathogenicity of these candidate variants and to discern which of them are *de novo* mutations and which are inherited cases with incomplete penetrance in prior generations or with a large pedigree of female-to-female transmission, as applicable. Of note, the known molecular mechanisms for some genes may provide guidance on the most likely option, as occurs in cases with mutations in the *PRPF31* gene, one of the most prevalent genes in this and other studies^17^, commonly showing incomplete penetrance^18^. These results emphasize the role of genetic testing in genetic counseling and clinical management, and highlight the need to give greater consideration to minority patterns of inheritance, which in turn have a high family impact. In addition, the identification of the underlying genetic defect may be decisive to future applications of gene-based therapies^19^ and genotype-phenotype correlations will be better defined as genetic testing become widespread.

The relative high frequency of RP has made it the most widely studied IRD, which has led to traditionally classify as RP all cases capable of being although clinical features were also consistent with other conditions. This, together with overlapping phenotypes, explains somehow the high proportion of clinical refinements obtained in this study (30.3%), although we expect it to lessen over time due to the progress made in ophthalmic techniques and in the knowledge of different IRD. Nevertheless, the extreme clinical and genetic heterogeneity of IRD will remain a problem to establish accurate genotype-phenotype correlations. Despite this, the genetic data obtained here allowed us to correlate the age of onset of symptoms with the causative gene and the mode of inheritance. As a result, while early-onset forms of the disease were the most common in all inheritance modes, only autosomal dominant cases showed a large group of late-onset forms. These results were in accordance with previous published studies in which autosomal dominant cases were considered milder forms of RP with later onset and tendency to retain good visual acuity^20^.

Remarkably, these results also allowed us to propose that the development of the disease depends on the type of causative mutations. We have observed that although most of pathogenic variants were missense, they were not randomly distributed among different genes and modes of inheritance. For example, *PRPF31* and *RPGR* did not show missense mutations and presented a clear preference for splicing and stop-gained mutations, respectively, which is in accordance with previous studies^17^,^21^. Moreover, genes involved in more than one mode of inheritance also showed different proportion for each type of mutations. For instance, synonymous and missense variants were predominantly associated with autosomal dominant forms implicating *PROM1* and *RP1* genes, whereas splicing and nonsense mutations were primarily related to autosomal recessive cases. Although the mechanisms underlying the effect of each mutation on the aetiology of IRD are still unknown, we suggest that variants resulting in prematurely truncated proteins, or transcripts degraded by the NMD (*Nonsense-mediated mRNA decay*) pathway, were associated with a loss of protein function leading to recessive forms of IRD, while a priori less damaging mutations may result in a gain of function of the protein with further deleterious effects^22^.

Furthermore, we found that *USH2A* was the most frequently mutated gene, which is in line with previous reports^23^. This gene is commonly associated with Usher syndrome and non-syndromic RP but, after clinical reassessment, two solved cases harbouring *USH2A* mutations presented clinical characteristics resembling CRD. This association has been previously reported^24^, expanding the spectrum of phenotypes associated with *USH2A* mutations ranging from syndromic and nonsyndromic RP to isolated CRD. Likewise, four additional genes, *CEP290, PDE6B, PROM1,* and *GUCY2D,* were suggested as causative in cases with clinical features not previously associated with them (#22, #35, #39, #83, #112 and #119) but further analysis will be required to confirm the segregation of the variants with the phenotype, the initial clinical diagnosis and the novel genotype-phenotype correlations. Moreover, regarding genes with both dominant and recessive alleles, the absence of family segregation does not allow us to rule out the possibility that the disease in *a priori* dominant cases is inherited as a recessive trait with a second mutation in an unanalyzed region. On the other hand, our results support previously reported genotype-phenotype correlations such as the involvement of the gene *ZNF408* in RP^25^.

Although most causative mutations associated with IRD are extremely rare, certain variants are remarkably recurrent. An example is the p.C759F mutation of *USH2A*, found in a total of 5 cases in this study. While this mildly deleterious variant is widely associated with non-syndromic RP in heterozygosis^12^, another *USH2A* frequent change, the c.2299delG mutation, causes mainly Usher syndrome^26^. However, the two cases harbouring this heterozygous frameshift mutation showed typical RP without hearing loss (#66 and #69), which may indicates low pathogenicity of the second heterozygous missense variant (p.P412R and p.R303H, respectively). In this regard, genetic findings of this study allowed us to consolidate the association between a specific mutation and a given phenotype. On one hand, the only two cases with LCA caused by *CRB1* (#24 and #37) shared the same heterozygous mutation (p.I205fs) which indicates the correlation of this variant with this severe phenotype. This is noteworthy because *CRB1* mutations lead to a highly variable spectrum of clinical phenotypes but no genotype-phenotype correlations has been established except that *CRB1* null mutations may be over-represented in LCA cases^27^. Similarly, family #68 was found to carry the heterozygous c.316G > A *RHO* mutation which was identified as causal of sector RP as previously described^28^.

Importantly, these results allowed us to uncover interfamilial phenotypic variability under the same pathogenic allele in two cases. First, one of the most common *CERKL* mutations (p.R257*) was detected in three unrelated families but only one (#53) showed macular involvement. Although *CERKL* mutations are widely associated with both typical RP and CRD^29^, further studies will be needed to elucidate the cause of this interfamilial variability. We hypothesize that additional genetic and environmental modifiers may be modulating the development of the disease. Secondly, a previously STGD-associated *ABCA4* mutation (p.R1129L)^30^ was found in a family clinically classified as RP (#84). No clinical features of STGD were found after clinical reassessment but data reanalysis showed a nonsense *RP1* mutation in heterozygosis (p.R396*) that could be acting as a phenotype modifier, indicating a potential epistatic effect of *ABCA4* and *RP1* mutations.

In summary, properly perform genotype-phenotype correlations is repeatedly hampered due to the extreme heterogeneity of IRD but a suitable approach may greatly facilitate this arduous task. This is even more important when it comes to simplex cases in which the mode of inheritance is not inferred by the pedigree and the genetic diagnosis is critical to estimate the family impact of mutations and to take appropriate actions. This study has further demonstrated that combining both a population specific panel design and the prioritization of variants using local genetic data, allow obtaining higher diagnostic accuracy. Moreover, our results reinforced the idea that the most common mode of inheritance of simplex RP cases is autosomal recessive while at the same time it has revealed that X-linked and autosomal dominant forms are probably being underestimated with all implications this entails.

## Methods

### Subjects and Clinical Data

Our current cohort of study involved 108 unrelated Spanish families, comprising 106 sRP cases with no known mutations and two genetically solved adRP cases included as positive controls of mutations^10^,^11^. Additionally, our study cohort included 118 samples from healthy relatives belonging to 42 families, in case segregation studies of candidate variants were needed.

The study was carried out in accordance with the tenets of the Declaration of Helsinki and all experimental protocols were approved by the Institutional Review Boards of the University Hospital Virgen del Rocio (Spain). Prior to analysis, written informed consent was obtained from all participants or their respective legal representative.

Clinical diagnosis of RP was based on a full ophthalmic examination, fundus photography, perimetry, colour vision, full field and multifocal electroretinography (ERG). RP was defined as bilateral visual loss, initial hemeralopy, restriction of visual field, gradual increased bone spicule pigmentation, attenuation of retinal vessels, waxy disc pallor and reduced or extinguished ERG. Genomic DNA of all subjects was isolated from peripheral blood using standard protocols and its integrity was verified using both the fluorometric dsDNA quantification and agarose gel electrophoresis.

Additionally, we have used the Spanish population variant server web page (CIBERER Spanish Variant Server, CSVS, publicly available http://csvs.babelomics.org/), that contains population frequency information from the whole exome sequencing of 267 unrelated individuals, representative of the healthy Spanish population (Medical Genome Project, MGP)^31^.

### Capture panel design

A custom IRD panel was developed using SeqCap EZ Choice System (Roche, NimbleGen, Madison, WI) to cover all exons plus 25 bp of intronic flanking regions of 68 genes, among them 65 known to be associated with monogenic retinal disorders in Spanish population and 3 candidate genes. Additionally, three deep intronic regions of *USH2A, CEP290* and *OFD1* genes, containing known pathogenic mutations, were also covered. The genes were selected and compiled from literature or databases as previously described^11^. The probes covered a total of 1,245 regions and the entire custom design spanned 369,318 bp (panel details available upon request).

### DNA library preparation and targeted sequencing

Genomic DNA (gDNA; 1 μg) was sheared using Covaris S220 sonicator to achieve target peak of 180 to 220 bp (SonoLab 7.2 settings: Peak Incident Power, 175 W; Duty Factor, 10%; cycles per burst, 200; Temperature, 7 °C Duration, 160 seconds) (Covaris, Woburn, MA). Library preparation was performed according to the double capture manufacturer’s protocol (NimbleGen SeqCap EZ Library Double Capture (version 4.2)). The library pooling guideline was followed to capture 7 samples at once (“Multiplex DNA Sample Library Pool”). After amplification, the index-tagged libraries were then quantified using the Agilent 2100 Bioanalyzer (Agilent Technologies), qPCR and fluorimetric measurements. The library peak size was in the range of 150 to 500 bp. Finally, multiplexed libraries were pooled (2 × 7; 14 samples per run) and diluted to 2 nM for sequencing on Illumina’s MiSeq instrument using a v2 (300 cycles) reagent kit.

### Sequence data analysis

Results were analyzed using our validated data analysis pipeline^32^ with some modifications. Sequence reads were aligned to the reference human genome sequence (hg19) by using Burrows-Wheeler Aligner (BWA, version 0.7.12). The coverage and the percentage of reads on target were analyzed using the BEDtools package. SNVs and indels were called using GATK software (version 1.4) and filtered to eliminate low quality SNVs (coverage <20x and strand bias). Remaining variants were functionally annotated by ANNOVAR^33^ and those with a MAF higher than 0.015 in at least one of the searchable databases (dbSNP, 1000 Genomes, EVS, ExAC) were excluded. Additionally, the presence of all candidate variants was checked in the MGP 267 unrelated individuals, representative of the healthy Spanish population^31^. On the other hand, as previously described^11^, we used the Surecall software (Agilent) with the “pair analysis” option for the identification of potential copy number variations (CNVs) and to discard hemizygosis in patients with mutations detected in homozygosis in which family segregation could not be performed.

### Pathogenicity assessment

Sequence variants considered to be likely disease causing mutations in this study were: (i) those previously documented to be pathogenic in the literature, (ii) large deletions and duplications (CNVs), (iii) novel nonsynonymous variants, (iv) nonsynonymous variants that are not novel but its allele frequency retrieved in any of the public exome databases (such as 1000 genomes project, ExAC, EVS and CSVS) is <0.015 and there are no homozygous/hemizygous carriers, (v) synonymous variants predicted to affect splicing process.

Once the list of candidate variants was prioritized, we evaluated the association of these mutations with the particular phenotype of each individual considering the genotype-phenotype correlations described in the bibliography, entries listed in the OMIM database and expert opinion. Clinical features of patients with mutations in genes not classically associated with RP were reviewed in order to reassess initial diagnosis.

The pathogenicity of novel non-synonyms SNVs was predicted by PolyPhen-2 (http://genetics.bwh.harvard.edu/pph2/) and SIFT (http://sift.bii.a-star.edu.sg) algorithms. Additionally, to estimate the effect of synonyms or intronic mutations on the splicing process we used NNSPLICE (http://www.fruitfly.org/seq_tools/splice.html), Mutation Taster (http://www.mutationtaster.org/), Human Splicing Finder (HSF; http://www.umd.be/HSF) or NetGene2 (http://www.cbs.dtu.dk/services/NetGene2/). The nomenclature of variants was adjusted to the Human Genome Variation Society (http://www.hgvs.org) guidelines using Mutalyzer (http://www.LOVD.nl/mutalyzer).

In cases where a single heterozygous pathogenic mutation was found in a gene linked to an autosomal recessive trait, we evaluated the implication of a complementary rare intronic or synonymous variant in the same gene explaining the phenotype. In cases where no causal mutation was found, we manually searched for regions with unusually coverage 0x indicating a possible homozygous or hemizygous deletion.

### Validation of candidate variants and Segregation analysis

Sanger sequencing was performed to validate all the pathogenic and likely pathogenic sequence variants. To further determine the impact of candidate variants, we conducted the segregation analysis in available family members. Specifically in 21 families (Table 1), samples from 62 additional members were used to verify segregation of the sequence alteration with the phenotype by conventional Sanger sequencing according to the manufacturer’s protocols (3730 DNA Analyzer, Applied Biosystems, USA) (Primer sequences and reaction conditions are available upon request).

Furthermore, exon deletions and duplications were validated by multiplex ligation dependent probe amplification (MLPA), specifically SALSA MLPA probes P322 and P361 were used following manufacturer’s recommendations (MRC Holland, Amsterdam, The Netherlands). Additionally, the validation of the large deletion comprising *OFD1* and *RS1* (Xp22.2–22.13), in which commercial MLPA was not available, was performed using a qPCR assay (primers available upon request). The qPCR was performed on an ABI Prism 7500HT Fast Real-Time PCR System with SYBR Green (Bio-Rad, USA).

## Additional Information

**How to cite this article**: Bravo-Gil, N. *et al*. Unravelling the genetic basis of simplex Retinitis Pigmentosa cases. *Sci. Rep.* **7**, 41937; doi: 10.1038/srep41937 (2017).

**Publisher's note:** Springer Nature remains neutral with regard to jurisdictional claims in published maps and institutional affiliations.

## Supplementary Material

Supplementary Information

## Figures and Tables

**Table 1:** Causal and likely causal mutations identified in the 68 characterized RP cases, including 2 positive controls and 66 sRP individuals.

ID N°	Gene	Allele 1			Allele 2			Family Segr. (Size)
		cDNA	Protein	Ref.	cDNA	Protein	Ref.	
*Mutations in autosomal recessive genes*								
4	*ABCA4*	c.52C>T	p.R18W	34	c.1222C>T	p.R408*	35	Yes (3)
84	*ABCA4*	*c.3386G*>*T*	p.R1129L	30	c.3386G>T	p.R1129L	30	NA
51	*BBS2*	c.1176_1184del	p.N393_T395del	This study	c.1175G>A	p.G392E	This study	NA
100	*C2orf71*	c.3099_3100insCAGG	p.V1034fs	ExAC	c.3099T>C	p.P1033P	ExAC	NA^(†)^
27	*CEP290*	c.4393C>T	p.R1465*	36	c.4705-1G>T	splice site	4	Yes (6)
61	*CEP290*	c.6604delA	p.I2202fs	37	c.6604delA	p.I2202fs	37	Yes (3)
93	*CEP290*	c.7328_7332delAGAAG	p.E2443fs	38	c.2991+1655A>G	deep intronic	39	NA^(†)^
119	*CEP290*	c.4028delA	p.K1343fs	37	c.2929A>G	p.R977G	ExAC	NA
2	*CERKL*	c.769C>T	p.R257*	40	c.769C>T	p.R257*	40	Yes (4)
53	*CERKL*	c.769C>T	p.R257*	40	c.769C>T	p.R257*	40	NA
55	*CERKL*	c.769C>T	p.R257*	40	c.769C>T	p.R257*	40	NA
14	*CRB1*	c.2227delG	p.V743fs	41	c.3299T>C	p.I1100T	42	Yes (3)
24	*CRB1*	c.613_619del	p.I205fs	43	c.2843G>A	p.C948Y	44	Yes (3)
37	*CRB1*	c.613_619del	p.I205fs	45	c.498_506del	p.I167_G169del	45	NA
44	*CRB1*	c.2290C>T	p.R764C	44	c.2290C>T	p.R764C	44	NA
45	*CRB1*	c.848+1G>A	splice site	This study	c.848+1G>A	splice site	This study	NA
85	*CRB1*	c.4006T>A	p.L1336M	This study	c.4082T>A	p.L1361Q	This study	NA
56	*EYS*	c.35T>C	p.M12T	46	c.35T>C	p.M12T	46	NA
115	*GPR98*	c.13757A>T	p.E4586V	CSVS	c.7176C>T	p.S2392S	47	NA^(†)^
12	*GUCY2D*	c.3119G>A	p.R1040Q	This study	c.3119G>A	p.R1040Q	This study	Yes (4)
60	*MYO7A*	c.1993A>G	p.I665V	This study	c.4008delG	p.E1337fs	This study	Yes (5)
23	*PDE6B*	c.385G>A	p.E129K	This study	c.385G>A	p.E129K	This study	Yes (4)
81	*PDE6B*	c.984delG	p.V329fs	This study	c.984delG	p.V329fs	This study	NA
122	*PROM1*	c.1984-1G>T	Splice site	48	c.1984-1G>T	splice site	48	Yes (4)
95	*RDH12*	c.278T>C	p.L93P	49	c.464C>T	p.T155I	50	NA
10	*RP1*	c.1625C>G	p.S542*	54	c.5881C>T	p.Q1961*	ExAC	Yes (3)
49	*RP1*	c.1625C>G	p.S542*	54	c.C1625G	p.S542*	51	NA
17	*USH2A*	c.1391G>A	p.R464H	This study	c.13822C>T	p.R4608*	52	Yes (5)
36	*USH2A*	c.2276G>T	p.C759F	53	c.908G>A	p.R303H	54	Yes (3)
46	*USH2A*	c.2276G>T	p.C759F	53	c.12294+1G>A	splice site	This study	NA
66	*USH2A*	c.2299delG	p.E767fs	55	c.908G>A	p.R303H	54	NA
69	*USH2A*	c.2299delG	p.E767fs	55	c.12371C>G	p.P4124R	This study	NA
71	*USH2A*	c.7525C>T	p.R2509W	56	c.13576C>T	p.R4526*	57	NA
73	*USH2A*	c.2276G>T	p.C759F	53	c.920_923dup	p.H308fs	58	NA
74	*USH2A*	c.2276G>T	p.C759F	53	c.4645C>T	p.R1549*	59	NA
79	*USH2A*	c.12575G>A	p.R4192H	60	c.1841-2A>G	—	42	NA
87	*USH2A*	c.2276G>T	p.C759F	53	c.13808A>C	p.H4603P	This study	Yes (5)
103	*USH2A*	c.11156G>A	p.R3719H	61	c.12569T>A	p.V4190E	This study	NA
108	*USH2A*	c.908G>A	p.R303H	54	c.10403C>T	p.P3468L	This study	NA
114	*USH2A*	c.10712C>T	p.T3571M	62	c.10712C>T	p.T3571M	62	NA
101	*ZNF408*	c.1342C>T	p.R448C	ExAC CSVS	c.1572C>A	p.Y524*	This study	NA
*Mutations in autosomal dominant genes*								
7 C+	*PRPF8*	c.6994dupG	p.D2332fs	11				Yes^(#)^
8 C+	*RHO*	c.(937–2_944)del	Splice site	12				Yes
15	*RHO*	c.403C>T	p.R135W	63				Yes^(#)^ (6)
68	*RHO*	c.316G>A	p.G106R	64				NA
33	*CRX*	Deletion ex3–4: c.(100+1_101–1)_(*1097_?)del	Unable to predict	11				NA
48	*FSCN2*	c.908G>A	p.C303Y	ExAC				NA^(†)^
39	*GUCY2D*	c.160T>A	p.F54I	This study				NA^(†)^
83	*GUCY2D*	c.1912C>A	p.L638M	This study				NA^(†)^
35	*PDE6B*	c.754G>A	p.D252N	This study				NA^(†)^
112	*PDE6B*	c.263A>G	p.Q88R	This study				NA^(†)^
102	*PRPF31*	c.238+1G>A	Splice site	This study				NA^(†)^
104	*PRPF8*	c.6926A>G	p.H2309R	65				NA^(†)^
107	*RP1*	c.4780T>C	p.Y1594H	This study				NA^(†)^
50	*TOPORS*	c.2524dupA	p.T842fs	This study				NA^(†)^
22	*PROM1*	c.1551A>G	p.E517E	This study				Yes^(†)^ (4)
91	*PROM1*	c.1584G>A	p.L528L	This study				NA^(†)^
76	*PRPF31*	c.789G>A	p.S263S	CSVS				NA^(†)^
97	*PRPF31*	c.417C>A	p.V139V	This study				NA^(†)^
*Mutations in X-Linked genes*								
29	*RPGR*	c.739C>T	p.Q247*	This study				Yes (4)
42	*RPGR*	c.888_889delAA	p.I297fs	66				NA
59	*RPGR*	c.2257_2260del	p.G753fs	67				Yes (4)
11	*CHM*	Deletion ex3–9: c.(116+1_117–1)_(1244+1_1245–1)del	Unable to predict	This study				Yes (4)
94	*CHM*	c.315-2A>G	Splice site	This study				NA
65 (F)	*OFD1 RS1*	Large deletion Chr X (p22.2–p22.13)	Unable to predict	This study				NA
116(F)	*CHM*	c.238C>T	p.L80F	68				NA
70(F)	*RP2*/*RPGR*	*RP2*:c.995C>T	p.T332M	ExAC	*RPGR*: c.347_349delAAG	p.E116del	This study	NA^(†)^
78(F)	*RPGR*	c.619+1 G>T	Splice site	This study				Yes (2)

(C+): positive controls. CSVS: Variant present in the Ciberer Spanish Variant Server. ExAC: Variant present in the Exome Aggregation Consortium; EVS: Variant present in the Exome Variant Server; (F): Female (X-linked dominant); Size: Number of members of the family included the index patient. NA: No additional family members available; (#): de novo mutation; (†): Likely solved families, further studies needed.
