# Supplementary material for: Unravelling the genetic basis of simplex Retinitis Pigmentosa cases
Source: Sci Rep. 2017 Feb 3;7:41937. doi: 10.1038/srep41937 (PMC5291209; doi:10.1038/srep41937)
Supplement: Supplementary Information [file srep41937-s1.pdf]

SUPPLEMENTARY INFORMATION

Unravelling the genetic basis of simplex Retinitis Pigmentosa cases

Nereida Bravo-Gil[1,2#], María González-del Pozo[1,2#], Marta Martín-Sánchez[1], Cristina Méndez-Vidal[1,2], Enrique Rodríguez-de la Rúa[3,4], Salud Borrego[1,2] and Guillermo Antiñolo[1,2*]

[1] Department of Genetics, Reproduction and Fetal Medicine, Institute of Biomedicine of Seville, University Hospital Virgen del Rocío/CSIC/University of Seville, Seville, Spain.

[2] Centre for Biomedical Network Research on Rare Diseases (CIBERER), Seville, Spain.

[3] Department of Ophthalmology, University Hospitals Virgen Macarena and Virgen del Rocío, Seville, Spain.

[4] Retics Patologia Ocular. OFTARED. Instituto Salud Carlos III.

[#] These authors contributed equally to this work.

[*]Corresponding author:

Guillermo Antiñolo, MD, PhD

Department of Genetics, Reproduction and Fetal Medicine

University Hospital Virgen del Rocio

Av. Manuel Siurot s/n

41013, Seville. Spain

E-mail: guillermo.antinolo.sspa@juntadeandalucia.es

Manuscript # SREP-16-24192: Unravelling the genetic basis of simplex Retinitis Pigmentosa cases

Nereida Bravo-Gil, María González-del Pozo, Marta Martín-Sánchez, Cristina Méndez-Vidal, Enrique Rodríguez-de la Rúa, Salud Borrego and Guillermo Antiñolo.

Supplementary Table S1: Clinical findings in solved cases initially diagnosed of sRP. ad: Autosomal dominant; CRD: Cone rod Dystrophy; F: Female; LCA: Leber Congenital Amaurosis; M: Male; NA: Not available; RP: Retinitis Pigmentosa; RPE: Retinal pigment epithelium; STGD: Stargardt disease; xl: X-Linked.

ID Number	Sex	Age of onset	First visual symptom	Age at time of diagnosis	Symptoms	BCVA	Fundus	ERG	Additional findings	Clinical reassessment
2	F	30	Constriction of the visual field	35	Reduced visual field, night blindness, decreased visual acuity	0.2/0.3	Bone spicule pigmentation, pallor of the optic disc, narrowed vessels	NA	NA	RP
4	F	8	Decreased visual acuity	46	Central scotoma, night blindness, discromatopsy	Light perception	Pigment deposits in macula	NA	NA	STGD
10	F	10	Hemeralopy	14	Reduced visual field, night blindness, decreased visual acuity	<0.1/<0.1	Bone spicule pigmentation. Narrowed vessels. Macular atrophy.	Abolished	Farnsworth Test: not recordable (OD) and discromatopsy (OS)	RP
11	M	2	Hemeralopy	11	Reduced visual field, night blindness, photophobia	NA	NA	NA	NA	Choroideremia
12	F	Birth	Hemeralopy	< 1	Reduced visual field, night blindness, decreased visual acuity	NA	NA	NA	Nystagmus	LCA
14	F	4	Hemeralopy	4	Reduced visual field, night blindness, decreased visual acuity	0.1/0.05	NA	NA	NA	RP
15	F	5	Hemeralopy	16	Night blindness, decreased visual acuity, reduced visual field, photophobia	NA	NA	Abolished	NA	RP
17	M	11	Constriction of the visual field	25	Night blindness, decreased visual acuity, reduced visual field	NA	NA	NA	NA	RP
22	F	2	Constriction of the visual field	NA	Reduced visual field, night blindness, decreased visual acuity	NA	NA	NA	NA	RP
23	M	6	Hemeralopy	28	Night blindness, decreased visual acuity, reduced visual field	0.3/0.4	Bone spicule pigmentation,	Abolished	Cataract, suspected Meniere's disease	RP
24	M	3	Hemeralopy	6	Night blindness and reduced visual field	0.1/0.1	Macular atrophy	NA	NA	LCA
27	F	5 months	Unknown	NA	Intense photophobia (better scotopic vision)	NA	NA	NA	NA	LCA
29	M	2	Hemeralopy	11	Night blindness, decreased visual acuity, reduced visual field	0.7/0.7	NA	Abolished	NA	RP
33	M	NA	Unknown	NA	NA	NA	NA	NA	NA	LCA
35	M	50	Hemeralopy	52	NA	0.5/0.7	RPE changes in the periphery, peripapillary atrophy, salt and peeper fundus.	Decreased responses	NA	RP
36	F	NA	Unknown	NA	NA	NA	NA	NA	NA	RP
37	F	4	Constriction of the visual field	7	Reduced visual field, night blindness, decreased visual acuity	0.1/0.1	Pigment deposits in macula, bone-spicule pigmentation in the equatorial retina		Nystagmus, strabismus	LCA
39	M	23	Hemeralopy	24	Concentric reduction of the visual field, night blindness, decreased visual acuity	0.8/0.8	Salt and pepper retinopathy, RPE changes in the periphery, normal optic disc and blood vessels	NA	NA	RP
42	M	25	Hemeralopy	29	Reduced visual field (10º central), night blindness, decreased visual acuity	0.5/0.5	Bone spicule pigmentation, pallor of the optic disc, narrowed vessels	NA	NA	RP
44	F	First decade	Constriction of the visual field	NA	NA	NA	NA	NA	NA	RP
45	M	NA	Hemeralopy	NA	NA	0.32/0.25	Bone spicule pigmentation, narrowed vessels and pale optic discs	NA	Cataracts	RP
46	F	13	Hemeralopy	NA	Night blindness, reduced visual field	NA	Bone spicule pigmentation, narrowed vessels	Abolished	Photophobia	RP
48	M	Birth	Unknown	NA	NA	NA	NA	NA	Nystagmus, obesity	RP
49	F	Birth	Hemeralopy	11	Night blindness, reduced visual field	0.6/0.2	Bone spicule pigmentation (mild)	Decreased amplitudes	NA	RP
50	F	20	Hemeralopy	29	Night blindness, decreased visual acuity, reduced visual field	0.3/0.3	Bone spicule pigmentation	Decreased amplitudes	Cataracts	RP
51	M	2	Hemeralopy	5	Night blindness, reduced visual field	1/0.8	Bone spicule pigmentation and pale optic discs	Decreased amplitudes	NA	RP
53	F	24	Decreased visual acuity	NA	Night blindness, decreased visual acuity, reduced visual field	<0.05/<0.05	Typical of advanced RP with macular affectation	NA	Photophobia	CRD
55	M	Birth	Hemeralopy	NA	Total blindness	No light perception	Retinal dystrophy, bone spicule pigmentation	NA	NA	RP
56	F	37	Hemeralopy	44	Reduced visual field, night blindness, decreased visual acuity	0.6/0.5	RPE changes in the periphery, perifoveal atrophy, bone-spicule pigmentation in the equatorial retina, pallor of the optic discs	Abolished. Delayed visual evoked potentials	NA	RP
59	M	Birth	Hemeralopy	16	Night blindness, decreased visual acuity, reduced visual field	0.3/0.2	Bone spicule pigmentation, narrowed vessels and pale optic discs	Abolished	Myopia	RP
60	M	18	Constriction of the visual field	NA	Night blindness, decreased visual acuity, reduced visual field	0.7/0.6	Optic disc pallor	NA	Cataracts, photophobia, hearing loss (supposedly after an ear infection)	USHER
61	F	NA	Unknown	NA	NA	NA	NA	NA	NA	LCA
65	F	1.5	Hemeralopy	2.5	Night blindness, decreased visual acuity, reduced visual field	NA	Tapetoretinal degeneration	NA	Strabismus, hypermetropia, astigmatism, photophobia	xlCRD

ID Number	Sex	Age of onset	First visual symptom	Age at time of diagnosis	Symptoms	BCVA	Fundus	ERG	Additional findings	Clinical reassessment
66	F	22	Hemeralopy	NA	Concentric reduction of the visual field (20º central), night blindness, decreased visual acuity, photophobia	0.8/0.8	Bone spicule pigmentation, pallor of the optic disc, narrowed vessels	NA	NA	RP
68	F	33	Hemeralopy	37	Night blindness	1/1	Bone spicule deposits predominant in the superior part of the retina resembling sector RP (inferior temporal sector free), narrowed vessels, normal macula.	NA	NA	RP (sector)
69	M	First decade	Hemeralopy	NA	Concentric reduction of the visual field (5º central), night blindness, decreased visual acuity	0.4/0.4	Bone spicule pigmentation, narrowed vessels and pale optic discs	NA	Post-traumatic hearing loss	RP
70	F	NA	Constriction of the visual field	24	Reduced visual field and visual acuity, night blindness	NA	Bone spicule pigmentation, narrowed vessels and pale optic discs	NA	NA	RP
71	F	34	Hemeralopy	43	Reduced visual field, night blindness, decreased visual acuity, photophobia	0.1/0.1	RP sine pigmento', narrowed vessels	Decreased amplitudes	NA	RP
73	M	First decade	Hemeralopy	NA	Concentric reduction of the visual field, night blindness, decreased visual acuity	0.7/0.8	Increase of the brightness of internal limiting membrane. Bone spicule pigmentation outside the vascular arcade	Abolished	NA	RP
74	M	First decade	Hemeralopy	NA	Reduced visual field, night blindness, decreased visual acuity	NA	NA	NA	NA	RP
76	F	NA	Hemeralopy	NA	Concentric reduction of the visual field (8º central); Preserved visual acuity	NA	NA	NA	NA	RP
78	F	NA	Unknown	32	Night blindness, decreased visual acuity, reduced visual field	NA	NA	NA	Myopia	RP
79	M	40	Hemeralopy	NA	Night blindness, decreased visual acuity, reduced visual field	NA	NA	NA	Myopia, photophobia	RP
81	F	First decade	Hemeralopy	18	Night blindness, decreased visual acuity, reduced visual field	0.5/0.5	Bone spicule pigmentation, narrowed vessels and pale optic discs.	NA	NA	RP
83	M	First decade	Hemeralopy	NA	Night blindness, decreased visual acuity, reduced visual field	0.5/0.3	Tigroid fundus	NA	NA	RP
84	F	2	Hemeralopy	24	Night blindness, decreased visual acuity, reduced visual field	NA	NA	Abolished	Myopia, astigmatism	RP
85	M	12	Hemeralopy	40	Night blindness, decreased visual acuity, reduced visual field	NA	NA	NA	NA	RP
87	M	40	Hemeralopy	43	Night blindness, decreased visual acuity, reduced visual field	0.3/0.2	Bone spicule pigmentation, narrowed vessels and pale optic discs and macular atrophy	NA	Farnsworth Test: discromatopsy/ hearing loss at 49 years old	RP
91	F	32	Hemeralopy/ reduction of the visual field	36	Reduced visual field, night blindness	NA	Narrowed vessels, fundus albipunctatus, normal optic discs, drusen near the fovea	Abolished	Myopia, astigmatism, slow progression, cataract	Maculopathy
93	M	First decade	Constriction of the visual field	8	Night blindness, reduced visual field, photophobia, decreased visual acuity	NA	Bone spicule pigmentation, narrowed vessels and pale optic discs	NA	NA	LCA
94	M	11	Hemeralopy	12	Night blindness, constriction of the visual field, decreased visual acuity	NA	RPE atrophy	Abolished	Myopia magna	Choroideremia
95	F	1	Constriction of the visual field	First decade	Night blindness, decreased visual acuity, reduced visual field	0.2/0.1	Bilateral macular atrophy	NA	Strabismus since 9 months of age, nystagmus	LCA
97	F	13	Decreased visual acuity	29	Night blindness, reduced visual field, photophobia, decreased visual acuity	NA	Pallor of the optic discs, narrowed vessels, bone spicules in the periphery, peripappilary atrophy with relatively preserved macula	NA	Nystagmus	RP with early macular involvement
100	F	25	Unknown	NA	NA	NA	NA	Abolished	NA	RP
101	M	7	Constriction of the visual field	NA	Night blindness, reduced visual field, decreased visual acuity	NA	NA	NA	Cephalea	RP
102	F	8	Constriction of the visual field	29	Night blindness, decreased visual acuity, reduced visual field	NA	NA	NA	NA	RP
103	F	11	Decreased visual acuity	27	Night blindness, decreased visual acuity, reduced visual field	NA	NA	NA	Photophobia, myopia, astigmatism	CRD
104	F	17	Hemeralopy	21	Night blindness, reduced visual field	NA	NA	NA	Astigmatism	RP
107	M	25	Hemeralopy	25	Night blindness, decreased visual acuity, reduced visual field	NA	NA	NA	Photophobia	RP
108	M	25	Hemeralopy	39	Night blindness, decreased visual acuity, reduced visual field	NA	NA	NA	Cataracts	RP
112	M	17	Decreased visual acuity	NA	Night blindness, decreased visual acuity, reduced visual field	NA	NA	NA	NA	RP
114	F	19	Decreased visual acuity	50	Night blindness, decreased visual acuity, reduced visual field	0.6/0.5	NA	NA	NA	CRD
115	F	17	Hemeralopy	23	Night blindness, decreased visual acuity, reduced visual field	NA	NA	NA	Hearing loss	USHER

ID Number	Sex	Age of onset	First visual symptom	Age at time of diagnosis	Symptoms	BCVA	Fundus	ERG	Additional findings	Clinical reassessment
119	M	20	Constriction of the visual field	25	Reduced visual field, photophobia	NA	NA	NA	NA	RP
122	M	NA	Unknown	NA	NA	NA	NA	NA	NA	STGD
116	F	14	Hemeralopy/ Decreased visual acuity	NA	Night blindness, reduced visual field, decreased visual acuity, color vision alteration	NA	Atypical fundus. Pigment deposits within temporal superior vascular arcade, yellowish waxy optic disc, inferior macular drusen, posterior pole chorioretinal atrophy, discrete pigmentary deposits	NA	Strabismus, cataracts	Choroideremia

Manuscript # SREP-16-24192: Unravelling the genetic basis of simplex Retinitis Pigmentosa cases

Nereida Bravo-Gil, María González-del Pozo, Marta Martín-Sánchez, Cristina Méndez-Vidal, Enrique Rodríguez-de la Rúa, Salud Borrego and Guillermo Antiñolo.

Supplementary Table S2: Unsolved cases carrying one likely pathogenic allele. Allele frequency data derived from ExAC (The Exome Aggregation Consortium). ESCS: Enhanced S-cone Syndrome; FFM: Fundus flavimaculatus; LCA: Leber Congenital Amaurosis; RP: Retinitis pigmentosa; STGD: Stargardt disease; VUS: Variant of unknown significance.

ID Number	Phenotype	Gene	Exon	cDNA	Protein	Status	Reference	Allele frequency	Interpretation
113	RP	*ABCA4*	42	c.5882G>A	p.G1961E	Het	Cella et al., Exp Eye Res (2009). rs1800553	0.005054	Pathogenic STGD
89	RP with early macular involvement	*ABCA4*	43	c.5908C>T	p.L1970F	Het	Rozet el al., Eur J Hum Genet. (1998). rs28938473	0.002925	Pathogenic Late-onset FFM
90	RP with early macular involvement	*ABCA4*	45	c.6148G>C	p.V2050L	Het	Allikmets et al., Nat Genet (1997). rs41292677	0.002752	Pathogenic| unknown
98	RP	*ABCA4*	45	c.6148G>C	p.V2050L	Het	Allikmets et al., Nat Genet (1997). rs41292677	0.002752	Pathogenic| unknown
64	RP	*ABCA4*	46	c.6148G>C	p.V2050L	Het	Allikmets et al., Nat Genet (1997). rs41292677	0.002752	Pathogenic| unknown
43	RP	*GPR98*	39	c.8779G>A	p.V2927I	Het	This study. rs547397177	6.63E-05	VUS
96	Non syndromic RP	*BBS12*	2	c.1114_1115del	p.F372fs	Het	This study	5.78E-05	Likely pathogenic
110	LCA	*CEP290*	25	c.2691A>G	p.I897M	Het	This study	2.11E-05	Likely pathogenic
28	RP	*CEP290*	30	c.3517C>A	p.Q1173K	Het	This study	3.78E-05	Likely pathogenic
26	RP	*CNGB3*	14	c.1627G>A	p.V543I	Het	This study	1.66E-05	Likely pathogenic
57	RP	*EYS*	15-19	Deletion exons 15-19	Unable to predict	Het	Abd-El Aziz et al. (2008)	-	Pathogenic
19	RP	*EYS*	32-33	Deletion exons 32-33	Unable to predict	Het	Audo et al., Hum Mutat (2010)	-	Pathogenic
25	RP	*GPR98*	59	c.12208G>A	p.V4070I	Het	This study. rs200943280	9.19E-05	VUS
109	RP with early macular involvement	*NR2E3*	6	c.932G>A	p.R311Q	Het	Haider et al., Nat Genet. (2000). rs28937873	0.0003416	Pathogenic ESCS| Goldmann-favre syndrome
99	LCA	*PCDH15*	35	c.5296_5304dupGCTCCTCCT	p.A1766_P1768dup	Het	This study. rs397517466	0.000148	VUS
5	RP	*RP1*	4	c.6196_6198del	p.D2066del	Het	This study	-	Likely pathogenic
105	RP	*RPGRIP1*	10	c.1220dupA	p.Q408fs	Het	This study	-	Likely pathogenic
82	RP	*USH2A*	27	c.5363A>G	p.D1788G	Het	This study	8.24E-06	Likely pathogenic
